# Risk perception associated with an emerging agri-food risk in Europe: plant viruses in agriculture

**DOI:** 10.1186/s40066-022-00366-5

**Published:** 2022-03-13

**Authors:** Johny Hilaire, Sophie Tindale, Glyn Jones, Gabriela Pingarron-Cardenas, Katarina Bačnik, Mercy Ojo, Lynn J. Frewer

**Affiliations:** 1 School of Natural and Environmental Sciences, Newcastle University, Newcastle Upon Tyne, NE1 7RU UK; 2 National Agri-Food Innovation Campus, Fera Science Ltd (Fera), Sand Hutton, York, YO41 1LZ UK; 3 Department of Biotechnology and Systems Biology, National Institute of Biology (NIB), Večna pot 111, 1000 Ljubljana, Slovenia

**Keywords:** Consumer, Disease, Food security, Supply chain, Policy

## Abstract

**Background:**

Research into public risk perceptions associated with emerging risks in agriculture and supply chains has focused on technological risks, zoonotic diseases, and food integrity, but infrequently on naturally occurring diseases in plants. Plant virus infections account for global economic losses estimated at $30 billion annually and are responsible for nearly 50% of plant diseases worldwide, threatening global food security. This research aimed to understand public perceptions of emerging risks and benefits associated with plant viruses in agriculture in Belgium, Slovenia, Spain, and the UK.

**Methods:**

Online qualitative semi-structured interviews with 80 European consumers were conducted, including 20 participants in each of Belgium, Slovenia, the UK, and Spain. Microsoft Streams was used to transcribe the interview data, and NVivo was utilized to code the transcripts and analyze the data.

**Results:**

The results indicate that, while study participants were relatively unfamiliar with the plant viruses and their potential impacts, plant viruses evoked perceived risks in a similar way to other emerging risks in the agri-food sector. These included risks to environment and human health, and the economic functioning of the relevant supply chain. Some participants perceived both risks and benefits to be associated with plant viruses. Benefits were perceived to be associated with improved plant resistance to viruses.

**Conclusions:**

The results provide the basis for risk regulation, policy, and communication developments. Risk communication needs to take account of both risk and benefit perceptions, as well as the observation that plant viruses are perceived as an emerging, rather than an established, understood, and controlled risk. Some participants indicated the need for risk–benefit communication strategies to be developed, including information about the impacts of the risks, and associated mitigation strategies. Participants perceived that responsibility for control of plant viruses should be conferred on actors within the supply chain, in particular primary producers, although policy support (for example, financial incentivization) should be provided to improve their motivation to instigate risk mitigation activities.

## Background

Plant viruses represent an emerging agricultural risk, resulting in agriculture yield losses estimated at $30 billion per year worldwide [1–3], and account for almost 50% of emerging plant diseases globally [4–6], representing a threat to global food security [7]. In Europe, they are responsible for significant economic damage in a range of crops including vegetables, grains, and ornamentals [8]. Various plant viruses have been reported to have negative economic impacts. The most prevalent and economically impactful viruses internationally have been identified by [9] and include, tobacco mosaic virus, tomato spotted wilt virus, tomato yellow leaf curl virus, cucumber mosaic virus, potato virus Y, cauliflower mosaic virus, African cassava mosaic virus, plum pox virus, brome mosaic virus and potato virus X. There are various mechanisms by which plant viruses can be transmitted within food supply chains, including mechanically, on workers’ hands, footwear and clothing, or via contaminated equipment, or by transmission via various species of thrips, aphids, beetles, and whiteflies, contaminated seed, and pollen. This indicates that control at farm level may at least partially mitigate the risks posed [10]. Currently, there is no evidence for negative impacts of plants affected by plant viruses on human health [11], although there is potential for them to have negative impacts on ecosystem functioning [12]. There is a need for early identification of emerging agricultural and food risks in order to prevent them from resulting in negative health, environmental or economic impacts, and to ensure emerging risk identification can be embedded in the risk analysis process for food safety [13, 14]. In addition, citizen/consumer risk perceptions associated with affected vegetables, fruits and food ingredients may result in changes to consumption behaviours, with commensurate effects on the economic functioning of supply chains [15, 16]. At present, there is little understanding of public risk perceptions regarding emerging potential risks and benefits connected with plant viruses in agriculture. To our knowledge, the research presented here is the first that addresses public perceptions associated with plant viruses. We have conducted an analysis of Spanish, Belgian, Slovenian, and British consumers’ attitudes towards, and risk/benefit perceptions of, plant viruses. North, South, West and Central European countries were included in the research, as specific plant virus risks are identifiable in each of these biogeographic zones. For example, Luteoviridae in the UK causes crop losses, in cereals, legumes, cucurbits, sugar beet, sugarcane, and potato, amounting to between £40–60 million annually [17]. In Spain, Maize rough dwarf virus (MR) has been observed to infect 24% of commercial maize fields between 2001 and 2006, increasing to 80% in 2015 [18]. In Slovenia, Henbane Mosaic Virus (HMV) has been reported to significantly infect field tomatoes [19]. In Belgium, Pepino mosaic virus (PepMV) causes disease in tomato fruits and has been estimated to result in 50–60% of fruits being unmarketable [20] which significantly impacts the economic value of the crop [21]. In addition, these countries were included in the research because they are potentially differentiated in terms of socio-cultural differences in consumer perceptions, attitudes, and preferences towards foods, as well as which foods are consumed in local diets. It has been established that citizen/consumer risk perceptions may result in changes to consumption behaviours, with commensurate effects on the economic functioning of supply chains [15, 16]. At present, there is little understanding of public risk perception, and associated attitudes and the drivers of this, regarding the emerging potential risks and benefits connected with plant viruses in agriculture.

An emerging risk within a food supply chain can be defined as one that results “…*from a newly identified hazard to which a significant exposure may occur, or from an unexpected new or increased significant exposure and/ or susceptibility to a known hazard*” [22]. In the European Union (EU), plant health risk assessments are conducted by the European Food Safety Authority (EFSA), with similar assessment being carried out by government agencies in other European regions. The EFSA plant health (PLH) panel assesses whether a particular plant pest should be taken into account for inclusion in the EU lists of harmful organisms by carrying out pest categorizations, using pest risk assessments. The PLH panel produced more than 70 outputs of which six were risk assessments of plant pests in EU territory. The outputs published during the first two PLH Panel mandates (from June 2006 to June 2012) are based on affected crops (field crops, forestry, fruit crops, ornamentals, and vegetables). The 2012 result showed that field crops were those most negatively affected by plant viruses, followed by forests, ornamentals, and vegetables [23]. EFSA has the responsibility for identifying existing and emerging risks to plant health, the food and feed chain. Risk management decisions about plant health are taken by the European Commission and Council via the Standing Committee on Plant Health, composed of representatives of the various EU member states. Some National Plant Protection Organizations (NPPOs), such as The European and Mediterranean Plant Protection Organization (EPPO), which cover the whole of Europe, not just the EU, conduct both risk assessment and risk management of plant viruses [10].

Evidence is required by policymakers and industry stakeholders to improve risk control and mitigation associated with the challenges linked to emerging food safety hazards, and provide the tools for risk analysis, policy development and implementation [24, 25]. Emerging risks within the agri-food sector may be driven by (a combination of) socio-economic and biophysical factors, and represent a new threat, re-emergence of an existing threat, the unintended consequences of a planned activity within the supply chain, or emergence of an existing risk as a consequence of the emergence of new identification methods or knowledge [26, 27]. Various emerging risks can be identified in the context of the agri-food supply chain. These may be related to the unintended effects of technological innovation, for example in relation to data security and privacy in digital technologies [28]; the impacts of agricultural herbicides, pesticides, and chemicals, which affect soil and surface water negatively and represent potential threats to human and environmental health through accidental ingestion in foods and associated toxic affects [29]; emerging pathogens and toxins in the supply chain which may result in food-borne disease in humans and animals [30, 31]; geopolitical changes which may place pressure on resources required for risk assessment and management [32]; and climate changes which may affect crops and crop production throughout Europe as weather systems are affected and act as stressors to plants [33–35].

Slovic [36] has defined risk perception as the intuitive evaluation of the probability of a specific hazard occurring, and the extent and nature of people’s concerns about the potential consequences of the hazard should it occur. Research on risk perception over the past four decades has focused on understanding the relationship between psychological factors that define peoples’ risk perception, how this relates to their responses to risks across a broad range of domains, and the implications for both people’s individual behaviours and public policy [37]. The field of policy and decision-making in relation to risks is challenging because members of the public, experts, and policymakers may assess risks very differently [38]. People’s risk perceptions should be considered when developing effective risk communication strategies, in order to take account of their perceptions, concerns, and priorities as well as technical risk estimates [39, 40]. The public perception of risk is an important element of the socio-political context within which policymakers operate. At the same time, some social and political observers have suggested that society is becoming increasingly risk-aware [41]. Research into public risk perceptions associated with various naturally occurring and technical hazards has been applied with the aim of ensuring risk-related policies align with public views and priorities, and to ensure the efficacy of risk communication practices. It is known that a range of psychological factors influences people’s risk perceptions, including, their ethical concerns, their trust and distrust in scientific bodies, risk regulators and information providers, and the perception that they are excluded from the process of making decisions about risk management and regulation [42]. In addition, risk perceptions associated with a particular hazard may vary according to cultural context and demographic group membership [43], and these differences need to be understood in order to harmonize risk identification, regulation, and risk management, including for transboundary risks, including those in the agri-food sector [44].

Assessment of the risks associated with food safety may reflect an “objective” approach (using scientific analysis methodologies embedded in the natural sciences) or a “subjective” risk assessment. Societal responses to a specific hazard may also reflect a broader range of concerns, including ethical concerns and perceptions that exposure to the risk is involuntary or exposure to the hazard under consideration is uncontrolled, which may increase people’s concerns about the hazard [45]. What is acceptable in terms of risk exposure and management within one region or culture may not be acceptable within another, with concomitant implications for effective risk management and communication practices [46].

Emerging risks are potentially characterized by high levels of uncertainty, i.e. lack of precision about the probability of a risk occurring, and ambiguity or multiple interpretations of the risk [47]. Effective management of plant virus diseases is extremely important to farmers, horticulturists, foresters, manufacturers, and consumers [16]. However, plant viruses have infrequently been the focus of broader societal debate, in particular in relation to understanding public perceptions and associated attitudes. Although plant viruses are important plant pathogens, causing economic losses by reducing crop quality and quantity globally [7], studies on virus biodiversity suggest that plants infected with numerous viruses may not have any apparent ill effects on their hosts [3]. This is further complicated by various observations that some virus infections, especially in natural environments, can be beneficial or mutualistic to the infected plants [48], for example, conferring tolerance to abiotic stressors [49]. At present, plant viruses are not known to cause disease in humans [50].

Effective risk–benefit communication with the public is required in relation to emerging plant viruses, as both the availability and quality of food may be affected. In order to incorporate public concerns and values in risk communication activities, it is important to understand people’s perceptions of risk and benefits associated with plant viruses in agriculture. The aim of this research was to analyze societal perceptions of risks and benefits associated with plant viruses in the agricultural sector, in order to inform future policy and risk communication strategies associated with plant viruses.

The following research questions were addressed:

1. How do Belgian, Slovenian, Spanish and UK consumers perceive risks associated with plant viruses in agriculture?
2. What factors affect their risk perceptions?
3. Based on people’s preferences, can effective communication strategies to deliver risk–benefit messages concerning plant viruses in agriculture be developed?

## Methods

Ethical approval for the research was provided by Newcastle University Research Ethics Committee, approval number 750/2020 on 7th February 2020. Online semi-structured interviews were developed from the existing risk perception literature (see Table 1).

**Table 1:** Summary of interview questions and justification for their inclusion from the existing literature

	Description/rationale	References
*Interview questions*		
NB. All answers were “open ended”, and the interviewer asked study participants to explain their answers if relevant in the context of the interview		
*Risk perceptions*		
- What does the term “risk” mean to you? (Question number 1) - Are you aware of the difference between an emerging and existing risk? (Question number 2) - Are you aware of any risks in agriculture? Can you tell me more about these? (Question number 3) - Are you aware of any emerging risks in agriculture? Can you tell me more about these? (Question number 4) - When you think about risks in agriculture, can you think of some words to describe these? (Question number 5) - Do you think of anything else when you think about emerging risks in agriculture? (Question number 6)	*Risk perception* People’s attitudes toward different agri-food risks are determined by their risk perceptions. Risk perception influences the extent to which a risk is perceived to be acceptable or otherwise *Perceptual factors specifically associated with emerging agri-food risks* It is known that some risk perception l factors are more likely to be associated with emerging risks, or those risks perceived to be novel. Presenting a naturally occurring risk in an emerging or ‘crisis’ context may increase peoples fear, concern and risk perceptions People may perceive emerging agri-food risks to have characteristics in common with other potential hazards the agri-food sector, to which they make cognitive “reference” and which they judge to be risky in the same way	[51–54]
- Do you think plant viruses result in risks or provide benefits to agriculture? Or do you think plant viruses may have both positive and negative impacts? (Question number 9) - Can you describe any risks (if the interviewee identifies the risk question)? (Question number 10) - Can you describe any benefits (if the interviewee identifies the benefits)? (Question number 11) - Do you think there are any negative or positive impacts caused by viruses on the quality of foods produced on farms? (Question number 13) - Do you think there are any negative or positive impacts caused by viruses on the quantity of food produced on farms? (Question number 14)	It has been established that there is an inverse relationship between perceived risks and perceived benefits, across a range of different potential hazards, including within the agri-food domain	[55–57]
-What is the first thing that crosses your mind when you hear the term plant viruses? (Question number 8)	Affective responses may also be evoked by emerging or novel risks, such as fear, and is likely to be evoked on first presentation of information about an emerging risk	[53, 58]
Do you think there are any risks to… A. Plant health? B. Human health? C. Environmental health? D. The economy linked to farming and agriculture? (Question number 12)	Risk (and benefit) perceptions are linked to concerns which extend beyond impacts to human health but may include impacts on the economy or the environment	[59–62]
*Knowledge about (the risks of) plant viruses)*		
- Have you heard about viruses in crop plants before? If yes, can you tell me more about what you know about these? (Question number 7)	Lack of knowledge about an emerging risk *may* influence risk perceptions associated with that risk. It is important to understand what people know and understand in order to develop effective risk communication information	[39, 63, 64]
*Perceived responsibility for control*		
- Do you think farmers should control emerging risks associated with viruses in agriculture? Please explain your answer. (Question number 15) - Do you think the government should control emerging risks associated with viruses in agriculture? Please explain your answer. (Question number 16)		[65–67]
*Risk communication and trust*		
- Would you like more information about the risks and benefits of plant viruses? (Question number 17) How would you like to get this information? (If participants answered yes to question 17) -From whom would you like this information? ( If participants answered yes to question 17)	An effective risk communication development strategy should consider whom the public trust and the preferred media for delivering information	[68, 69]
*Who is likely to be the most affected by plant viruses*		
- Do you think farmers are worried about plant viruses? Why? (Question number 18) - Do you think your national government is worried about plant viruses? Why? (Question number 19) - Do you think people involved in the food chain (for example, those working in the retail sector) (Question number 20)	Emerging risks may differentially affect different actors in the supply chain. It is of interest to find out whom people believe are adversely affected, as this may influence their perceptions of the acceptability of a risk (or otherwise)	[39, 70]
*Policy interventions*		
- Should the government act to reduce negative impacts of plant viruses in the food supply chain? (Question number 21)	Evidence is required by policymakers with regard to challenges connected to emerging food safety hazards to ameliorate risk management, and deliver the tools for risk analysis, policy development and implementation. This should take account of citizen stakeholder preferences for risk policies in the policy development process. This includes whether or not the government should intervene, as well as how this should evolve into policy	[24, 25, 71]

The interviews were conducted online to overcome time and spatial constraints associated with qualitative research across multiple locations, and to overcome travel restrictions associated with the COVID-19 pandemic [72]. This methodology enabled interviewees to have the freedom to express their own opinions [73]. An informed consent form was signed by each study participant prior to the interviews commencing, and study participants were told that they could withdraw from the research and have their data destroyed at any time.

A semi-structured interview protocol was developed, aimed at understanding consumers’ attitudes towards emerging risks linked to plant viruses in agriculture, and to capture differences linked to cross-cultural factors and local or agronomic conditions in Belgium, Spain, Slovenia, and the UK (see Table 1). All study participants signed a consent form prior to the interview commencing. The research followed the standard process for conducting a semi-structured interview [74]. Synchronous online interviews [75], including video, text, and visual exchange were used. The discussion guide was available in French, English, Slovene, and Spanish, and was either translated from English by the recruitment agency “Team Search”, or, in the case of interviews in interviews conducted in Spain and Slovenia, project members who spoke Slovene and Spanish assisted with the Slovenia and Spanish translation and data collection. Participants were asked about their knowledge about plant viruses, and whether they though plant viruses result in risks and/or benefits to agriculture. A further question was asked regarding whether farmers, governmental agencies or both should control and mitigate the emerging risks associated with viruses in agriculture, and whether the government should act to reduce negative impacts of plant viruses in the food supply chain. Participants’ interest in receiving more information about the risks and benefits of plant viruses, and who among different stakeholders would be most negatively affected, was also included. Images of infected plants by viruses were shown to those who self-reported did not know anything about plant viruses.

### Sample

Twenty participants were interviewed in each of Belgium, Slovenia, the UK, and Spain. Seventy-nine out of 80 interviews were carried out via Zoom, and 1 via Microsoft Teams. The demographic profile of the participant sample is provided in Table 2. The thematic analysis (see “Results” section”) indicated that saturation had been reached at 20 interviews, and further data collection would have not yielded further results. Interviewees were selected through a social research agency “Team Search”, which recruited participants from a panel on the basis of socio-demographic characteristics, including gender, age, (over 18 years) and occupations. An additional requirement was that participants were primarily responsible for food purchases in their households. Each interview lasted between 30 min and 1 h and was electronically recorded and transcribed *verbatim*. All interviewees were provided with an incentive payment of €40 following their participation in the research, in accordance with standard remuneration determined by the research agency for research participants in Europe. All data were collected between September and November 2020.

**Table 2:** Summary of allocation of thematic codes (number of participants associated with each thematic code) by demographic groups

	Belgium		UK		Slovenia		Spain		Total
Gender	Female	Male	Female	Male	Female	Male	Female	Male	
Perceptions of risk and benefit associated with plant viruses									
Risk and benefit perception	5	5	7	5	4	6	5	5	42
Risk perception	8	2	4	4	4	6	5	5	38
Negative affect associated with the term virus									
Negative perceptions	0	0	3	2	0	1	2	0	8
Self-reported knowledge about plant viruses									
No knowledge	5	2	4	5	4	7	3	5	35
Very knowledgeable	6	3	5	1	4	4	4	3	30
Limited knowledge	2	2	1	2	0	0	3	1	11
Perceived responsibility for control of plant viruses									
Farmers	11	5	5	7	6	10	10	7	61
Government	6	7	5	3	7	5	7	3	43
Stakeholders perceived to be the most and the least affected by plant viruses									
Farmers, the most affected	12	6	7	7	6	10	9	9	66
National Government, the least affected	0	0	0	0	0	0	1	0	1
Participant interest in risk–benefit information about plant viruses									
Yes	13	7	9	8	7	10	8	9	71
No	0	0	1	0	1	1	2	0	1

### Procedure and analysis

Transcription of the interviews was conducted by the lead author, allowing the researcher to increase connectivity with the data [74], and to identify themes [76] relevant to the research questions [77]. A thematic analysis of the abstracted codes allowed identification of key ideas and procedures, as well as enabling comparison between study participants from different demographic groups and countries. Coding was conducted using NVivo 2020. Open coding, in which transcripts were carefully read, was applied to identify cross-cutting themes [78, 79]. When new categories no longer emerged, the coding process was finalized as saturation had been reached [80].

## Results

Six themes emerged from the interview transcripts: (a) perceptions of risk and benefit associated with plant viruses; (b) negative affect associated with the term virus; (c) self-reported knowledge about plant viruses; (d) perceived responsibility for control of plant viruses; (e) participant interest in risk–benefit information about plant viruses; (f) stakeholders perceived to be the most and the least affected by plant viruses. For brevity, the findings are summarized in Table 2. Exemplar quotes from the interviews, and the accompanying narrative explanation, are provided in Appendix. The presentation of the results reflects the stages of the thematic analysis process. The quotes’ structure is as follows: participant’s identification number, preceded by ‘P’, name of the country of origin, gender, and age band of the participant.

### a) Perceptions of risk and benefit associated with plant viruses

Plant viruses were perceived either as both risky and beneficial, or just risky. Where both risks and benefits were perceived as important, risks were perceived as being more heavily weighted in terms of attitudes than benefits. Risk perceptions focused on environmental, plant and human health risks, and economic problems and losses linked to diseased plants. Perceptions of benefit related to positive adaptations and resilience development of plants affected by viruses. Respondents thought that studying or researching plant viruses can be beneficial for the plants, as new knowledge can be generated, and findings could be used to mitigate risks associated with plant viruses.

### b) Negative affect associated with the term virus

The word virus was linked with negative associations and potentially evoked negative affective or emotional responses, including fear. This tended to be captured in initial participant responses.

### c) Self-reported knowledge about plant viruses

The majority of participants were not highly knowledgeable, about plant viruses. A minority of participants were able to provide a description about plant viruses, having either read about them or having seen plants infected by viruses. Plant viruses were viewed as causing “a disease” and were frequently described as something that attacks and kills plants. Participants who knew about plant viruses could explain how viruses are transmitted and elaborated on the difference between RNA and DNA viruses. The viruses described by participants included those affecting tomatoes, potatoes, and tobacco. The terms “dangerous”, “disease”, “damage plants”, “(negatively) affect plant growth”, “unhealthy”, and “change plant leaves’ colour” were the most frequently described first impressions respondents had of plant viruses. Participants with limited knowledge of plant viruses expressed this directly. This level of knowledge made it difficult for participants to describe what they understood about plant viruses. While they were aware of tomato and potato virus, study participants were unable to provide an explanation of how these viruses attack tomatoes and potatoes. While participants were aware of the existence of plant viruses and the threat they posed to plants, they were unable to articulate what these were in any detail. Some participants described only hearing the term viruses during the COVID-19 outbreak. When participants were shown images of plants infected by viruses, some expressed the view that the infection resulted from climate change. Participants were aware of other existing and emerging agricultural risks. The most frequently mentioned included pesticides, chemicals, GMOs, and links to climate change.

### d) Perceived responsibility for control of plant viruses

The view was expressed that farmers should proactively control the risks of plant viruses before these risks got out of control. Some participants expressed the view that better control of plant viruses resulted in healthier food for consumers. To protect plants and consumers, it was thought that farmers should learn and have knowledge of appropriate control measures. As part of this, farmers should exchange knowledge with other stakeholders in the agricultural sector and learn how to mitigate viruses infecting their crops. Effective control was seen as an advantage for both the farmer and the national economy. Participants also indicated that government institutional support should be provided to enable better control of plant viruses. Some potential control measures were described by participants, for example increased availability of high-quality seed to farmers non-chemical control of plant viruses was preferred by some participants. The government was viewed as having responsibility for implementing measures to reduce negative impacts in the food supply chain beyond the farm gate. Governmental actions to mitigate negative impacts in the food supply chain were described in terms of increased farmer empowerment via, for example, fiscal incentivization, or increasing research investments in projects aimed at reducing the impacts of plant viruses. Regulatory action to control or mitigate plant virus were also perceived to be a governmental responsibility, with some participants expressing the view that these should be established at the European level if possible. These regulations would guarantee that consumers have access to high-quality foods.

### e) Participant interest in risk–benefit information about plant viruses

Most participants indicated that they were interested in to receiving information about the risk and benefit associated with plant viruses. This was, first, because they wished to take informed decisions about purchases, second, because they wanted to know about the benefits of plant viruses, and third, because they were curious about these risks and benefits. Expressing views as the consumers of food products, participants also indicated that, they had a right to know about what they were consuming. Email was the preferred media for information provision given the fact that information could be delivered more easily, and the content of the information would be always available. TV was mentioned together with other ways of getting information and was viewed as a way of watching agricultural programmes. Participants’ preferred information source was the government or farmers.

### f) Stakeholders perceived to be the most and the least affected by plant viruses

Farmers were perceived by participants to be the stakeholders most likely to be negatively impacted by plant viruses. Participants were concerned about the impacts of plant viruses on their personal health as well as the potentially negative effects of farm-level mitigation strategies, in particular the use of pesticides and the potential of pesticide residues on foods to have negative impacts on health. Concerns were raised in relation to potential price increase of food as a result of plant viruses linked to reduced availability of products. It was argued that national government is the least worried stakeholder. Stakeholders involved in the middle part of the food supply chain were not perceived by participants to be concerned about plant viruses since they sell foods, and their interest is focused on profit. Participants were observed to exhibit more negative affective responses to plant viruses as the interviews proceeded, implying that provision of risk communication or information may produce an affective response, which may subsequently impact on how people make decisions about risks using heuristic or deliberative information processing [81]. That is, people may judge the risks and benefits of specific hazards by referring to the positive and negative feelings they associate with them. Experiencing negative affect in association with plant viruses will increase risk perception and reduce benefit perception.

### Demographics

The sample size is too small to provide a full comparative analysis, but a descriptive analysis of potential issues for further research is described here. Some trends within the data were identified (Table 2), but these need to be confirmed using a larger representative sample. Female, and younger participants more frequently reported that they were aware of plant viruses. Participants from Belgium self-reported being more informed about plant viruses than participants in the other countries. In relation to responsibility for control of viruses, female participants more frequently indicated that they perceived that farmer should be responsible for controlling the risks associated with plant viruses in agriculture. Spanish participants more, and UK participants least, frequently indicated that they felt farmers should have responsibility for controlling plant viruses. Overall, more male participants expressed the view that the government should act to reduce negative impacts in the food supply chain. In terms of differences between countries, equal number of participants in Belgium, Spain, and Slovenia, indicated that they thought the government should act to reduce negative impacts of viruses in the food supply chain. As for information provision, female participants expressed a greater preference for receiving information about the risks and benefits of plant viruses. Participants in Belgium more frequently indicated that they would like to receive information on the risks and benefits of plant viruses compared to participants in the other countries. Participants aged between 35 and 54 were most, and those over 55 least, likely to report that they perceived both risks and benefits to be associated with plant viruses, with participants older than 55 being least likely. Participants aged 18–34 more frequently, and those over 55 least frequently, reported risk perceptions associated with plant viruses. More respondents in the age group (18–34), and less in the age group 55+ indicated a preference for the government to act to reduce negative impacts in the food chain than participants in the other age bands.

## Discussion

The results suggest that research participants perceived plant viruses within the agri-food sector as an emerging risk. In terms of risk perception, emerging risks, which are frequently associated with high levels of uncertainty and ambiguity, may trigger high levels of concern, although this is not always the case [82]. The results suggest that people perceive risk to be associated with plant viruses, and indeed this is characterized by uncertainty and ambiguity, as well as the requirement for effective risk communication to be implemented. While participants were aware of existing and emerging risks in agriculture, citing examples such as climate change or genetically modified organisms (GMOs) used in the agri-food sector, the majority were not aware of plant viruses until they were enrolled in this research study. Some participants also identified potential benefits to be associated with plant viruses. This lends support to the idea that risks, the lack of risks in case of plant viruses not causing diseases in plants, and benefits, need to be considered in the process of risk analysis [16], as well as EFSA priorities regarding risk–benefit assessment [83]. Research into plant viruses suggests that many viruses identified in different plant hosts are not causing any symptoms in plant, nor causing diseases and therefore do not represent a risk per se, in terms of plant health or negative economic impacts given that risks and benefits need to be communicated as part of a transparent risk communication process, communication should also include messages about the lack of impact, whether positive or negative [84].

Research participants identified a range of benefits to agri-food production in relation to plant viruses, including increased resistance to disease and abiotic stressor resistance, although beneficial effects of plant viruses have been very poorly studied, and are unexploited in crops [39]. In this context, the need to allocate research funding to understand the impacts of plant viruses in the agri-food sector was recognized by participants in order to mitigate the potential environmental health issues associated with plant viruses, and the negative economic impacts on supply chain actors, in particular primary producers (farmers). Although there was some recognition within the participant sample that risk prevention and mitigation associated with plant viruses was a shared responsibility between government and farmers, the latter were perceived to have the primary responsibility for their control if this was required. As a consequence, it is necessary for a combination of regulatory actions and policy levers to be put into place and implemented to ensure this happens. For example, according to the research participants, national and European governments should fund farmers to control emerging risks associated with plant viruses in agriculture, ensure that timely risk identification and characterization practices are implemented where it is advantageous to reduce the spread viruses in the agri-food sector.

Risk communication is an integral part of the risk analysis process recognized by Food and Agriculture Organization (FAO) and the Codex Alimentarius [49]. The results of this research indicate that, in common with other (emerging) food risk issues, there is a need to develop and implement an effective risk communication strategy, ensuring that risk uncertainty is addressed together with timely updates about advances in scientific knowledge and what is being done to reduce this [85–87]. In this context, it may also be relevant to consider new forms of knowledge exchange (crowd sourcing, social media analytics). Crowdsourcing can positively contribute to risk assessments as it can help to collect huge amounts of data instantly, potentially cheaper than conventional approaches. Crowdsourcing therefore represents a channel for risk communication through sensitization and outreach. Crowdsourced approaches make risk assessment more inclusive, improving both the quality of the risk assessment, and increasing public confidence [88].

Although the participants in this research expressed preference for digital risk communication, it is also important to ensure that some members of society who do not have access to the internet and other digital communication systems such as mobile phones, and who at the same time may be particularly vulnerable to food risks, are not excluded from food safety risk communication, including in relation to plant viruses. “Traditional” risk communication media therefore need to be retained by communication practitioners.

This shows the importance of developing an effective communication strategy about the risks and benefits of plant viruses to address this lack of awareness. An effective risk–benefit communication strategy is needed, which builds on public concerns as well as supply chain risks. However, this may in itself generate an affective or emotional response, which will influence how people process information (Jin et al. submitted). In addition, the use of some mitigation strategies, including the application (e.g. “use of pesticides”) increased participant risk perceptions as the mitigation strategy was in itself perceived as risk [89, 90]. However, there are currently no antiviral agents to protect plants from viruses circulating in agroecosystems. Control measures focus on the selection of varieties that are resistant to viral diseases, the improvement of varieties and control of the number of insect vectors [91].

Consistent with previous research, participants expressed lack of trust in government and industry, in relation to their concerns about plant viruses. Given trust in institutions and information sources are important for determining public responses to risk communication [92], understanding risk perceptions and other concerns about plant viruses, and embedding these in risk communication at EFSA, or in national level communication in Europe, might increase trust in information sources. For example, some participants were concerned that plant viruses might have a negative impact on human health. It is currently considered that, unlike animal viruses, plant viruses cannot replicate in humans or other mammals, and therefore, plant viruses cannot cause disease in humans [51, 93]. There may therefore be a need for risk communication to emphasize the safety of plant viruses in regard to human health, including the counteraction of digital “fake news”, in particular in the post-COVID era where the public have been particularly sensitized to potential negative health impacts associated with the term virus.

## Conclusions, and suggestions for future research, and limitations

Participants across the UK, Spain, Slovenia, and Belgium perceived risks, and to some extent benefits to be associated with plant viruses. Most of the participants indicated that they were not familiar with, or knowledgeable about, plant viruses, which indicated that plant viruses represented an emerging risk as far as participants’ risk perceptions were concerned, and that any communication strategy needs to assume that issues to be discussed (such as uncertainty and what is being done to reduce this) plant viruses represent an emerging risk issue for the public. Primary producers (farmers) were perceived to have responsibility for prevention and mitigation of plant viruses in the agri-food sector, although the need to develop policy levers, such as incentives, and regulations, was perceived to be within the remit of government. For example, governments could develop policies to empower farmers with finance and training delivered via (e.g.) extension services to control the risks associated with plant viruses. As participants expressed some concern about the impacts of plant viruses on human as well as environmental health, information about plant viruses being safe for human health should be addressed in both digital as well as traditional media for risk communication. The perception that benefits as well as risks were associated with plant virus aligns with proposed changes from risk assessment to risk–benefit assessment proposed, inter alia, by agencies such as EFSA.

In terms of developing an effective risk–benefit communication strategy, it is important to note that people tend to weigh negative information as having a greater negative impact compared to the lack of, or beneficial impacts linked to benefit information. In other words, risk information will have a greater impact on human decision-making than benefit information [94]. Many participants in this research perceived both risk and benefits to be associated with plant viruses, although few were aware of plant viruses before recruitment. It would be valuable to conduct experimental research where risk and benefit content is varied in terms of the impacts linked to plant viruses and assess the impact on risk and benefit perceptions. As people are relatively unaware of plant viruses, the presentation of information about them might in itself evoke an affective response, which will in turn influence perceptions, and this potential influence needs to be incorporated into the experimental design of such research. There is some evidence of demographic and national differences in risk and benefit perceptions, but the participant sample is too small to confirm that these differences are significant. Larger quota sampled surveys including analysis of cross-cultural differences, are required. Further, given that the data for this research were collected during the pandemic crisis, it is uncertain as whether participants associated the term “virus” with more negative impact than prior to the COVID-19 outbreak. It is not clear whether risk perception attenuation (for example, linked to mitigation measures associated with the pandemic) might occur in the future, reducing at the same time risk perceptions associated with plant viruses. This will require additional research in the future.

Various study limitations have been identified. First, although there was some evidence that demographic and cultural differences influenced risk and benefit perception, the sample size was too small to allow a comparative assessment of whether these differences were significant, nor establish the causes. Given that there may be a need for a targeted communication strategy to be developed that focuses on people’s concerns as well as technical communication issues, a larger quantitative survey, or indeed more extensive qualitative research, might provide relevant evidence. Second, images of infected plants by viruses were shown only to participants who self-reported not being aware or knowledgably plant viruses. Exposure to images may have influenced perceptions within the group of participants who self-reported that they were familiar or knowledgeable about plant viruses.

## Data Availability

The data of this study are available upon request from the authors. The data are not publicly available due to privacy policies associated with the GDPR.

## Appendix

See Table

**Table 3:** Online interviews: themes supporting evidence and researcher interpretations of the data

Theme	Theme description	Example quotes from interview data	Researcher interpretation of the qualitative data
Perceptions of risk and benefit associated with plant viruses	Perceptions of both risk and benefit, and perception of solely risk associated with plant viruses	‘If it’s a good virus, maybe it can be positive, but 3/4 of viruses, I think they are rather negative’ (P4-Belgium-Female-35–54) ‘….it could potentially lead to benefits as well because I imagine that plants have to develop ways of withstanding the viruses, and hence it might lead to some positive changes where they kind of adapt to these new conditions, so I'm guessing that there could be potential benefits as well’ (P68-UK-Female-18–34) ‘I think on balance they're [the viruses] going to have negative impacts, but there will be some positives as well, because as new viruses attack plants, plants themselves will mutate ….and become stronger themselves, learning too, in a way, to get over what is happening to them’ (P77-UK-Female-55+) ‘The positive impact would be …researchers can sample and study them and can be better at preventing them’(P16-Belgium-Female-35–54) ‘Viruses are used to produce vaccines, and therefore we can do research on the virus to get some benefits…’ (P44-Spain-Female-55+) ‘These [plant viruses] are risks, we no longer know how to eat them when there is a virus and you get sick, probably if you don't know if we are not aware that these are viruses….’ (P2-Belgium-Female-55+) ‘If a plant is infected, it can cause diseases in some humans. So yes, it’s important to take that into account when we consume certain foods, we could be infected with the disease of the plant virus’ (P11-Belgium-Male-18–34)	The most frequently expressed perceived risks associated with plant viruses included those associated with the environmental, plant and human health risks, and economic problems and losses linked to diseased plants. Participants described scenarios where viruses can spread from one plant to another and negatively affect surrounding fields. Some participants believed that eating a plant affected by viruses may produce adverse effects on human health The most frequently expressed benefits were associated with the perception that plants exposed to viruses can get immunity to them; plants can become stronger; that the study of viruses can inform medical research; and that viral exposure can help crops to develop abiotic stress resistance linked to temperature, and drought tolerance
Negative affect associated with the term virus	Association of plant viruses with negative perceptions and affect	‘…. if I hear the word virus [it] is negative, that's going to be a bad thing. That's going to cause trouble. Whether it wipes out crops, and you know, people go hungry…’ (P62-UK-Female-35–54) ‘The name of virus means poison, so they are bad for everything, for humans, plants, and animals’ (P44-Spain-Female-55+) ‘I’d say I’ve become a lot more worried about anything involving the word virus. Over the past 6 months……’ (P75-UK-F-55+)	The word “virus” evoked negative affective responses for some participants
Awareness of plant viruses	The extent to which participants were aware of the existence and/or impacts of plant viruses	‘…I have heard about viruses. I have also seen some of the viruses ……pictures [of] how this looks and what it does to […] different plants, to the leaves, or to the whole plant or something that is growing, it’s dangerous’ (P40-Slovenia-Male-18–34) ‘You have viruses that create disease problems and those disease are difficult to treat’ (P1-BEL-Male-55 ) ‘A virus […] it’s built to survive and therefore it uses the cell of a plant, and it kills the host’ (P41-Spain-Male-18–34) ‘They [viruses] need to enter the cells, and for that normally they enter through insects or wounds and then they infect the plant. They are two kinds of virus, the ones that replicates through DNA and the ones that use RNA’ (P47-Spain-Female-35–54) ‘No, I know very, very little. I just know there can be viruses….’ (P3-Belgium-Female-18–34) ‘I have a very small amount of knowledge…Basic, very basic knowledge, other than living on a rural area where I live’ (P65-UK-Male-35–54) ‘The only thing is I started back when coronavirus basically started hitting the news, I guess […] about January, February time I basically put […] into a search engine about viruses. I can't remember exactly what came up […], all I can remember, very few plant viruses are harmful to the plant itself” (P72-UK-Male-55+)	The range of knowledge about plant viruses and their potential impacts (both negative and positive) varied considerably between participants, with some indicating that they were unfamiliar or unknowledgeable about their existence of their potential impacts, while others had more extensive knowledge about these issues
Perceived control of plant viruses	Identification of which stakeholders should have responsibility for controlling plant viruses	‘Yes. I think they [farmers] should control [plant viruses]…’ (P27-Slovenia-Male-18–34) ‘So, since it has to do with our health, it has to be very well controlled. …. It is the farmer’s job to do that, to make sure that the products he will sell will be good for people who will consume them and who will buy [the products]…’ (P3-Belgium-Female-18–34) ‘…. I think they [farmers] might not have enough information, or they should talk with people specialized in agriculture that can advise them about the causes and solutions and ways to prevent the appearance of viruses’ (P55-Spain-Male-55+) ‘Plant viruses are emerging risks, that’s why we have to control them. I think farmers should continue to control them. It's not just for the national economy, but for them too’ (P10-Belgium-Female-35–54) ‘Organizations can also help them to diagnose viruses and get some cure against the viruses’ (P33-Slovenia-Male-18–34) ‘One way to control them would be making sure that the seeds come from for a good place, …. to follow a quality control, like the meat’(P43-Spain-Female-35–54) ‘Farmers are struggling these days, and they got to try to combat plant viruses, but I see dangers with regard to what farmers will use to try to combat viruses, such as with chemicals and fertilizers. That might actually be worse than the virus itself” (P77-UK-Female-55+) ‘…they [the government] should give help like money or machinery to the farmers so they can control the virus’ (P54-Spain-Female-35–54) ‘They should be providing adequate funding so that science, so that scientists, botanists, and people at local levels can contribute and do what they can to avoid emerging issues with plant viruses…’ (P70-UK-Male-35–54) ‘The government… has a certain responsibility for the health of citizens. So …the government should be…, regulate, …and put in place rules to decrease the risk of viruses’ (P3-Belgium-Female-18–34) ‘Agricultural policy is more of the European domain anyway. I think certainly at the European level that measures must be taken….’ (P8-Belgium-Male-35–54)	Participants expressed the view that farmers should be responsible for controlling risks associated with plant viruses in agriculture, and that the government represents a key stakeholder who should act to reduce negative impacts associated with plant viruses in the food supply chain. This was explained as farmers being in close contact with their fields and these noticing problems first
Participant interest in risk–benefit information about plant viruses	The extent to which participants said they would like to have access to information on the risks and benefits associated with plant viruses	‘Yes, I would like to. It would help to make decision when I get fruit and vegetables’ (P41-Spain-Male-18–34) ‘Yes, I would like to. But specially about the benefits, because personally, I do not know any, but I know about loads of bacteria that are good and give us benefits. There must be a virus that gives some benefits’ (P42-Spain-Male-35–54) ‘Yes …I don't know at all what it is. I may have heard like that, once a virus, but plant viruses I don't know at all what it is so that it would be good to have a little information on this because nobody talks about it’ (P3-Belgium-Female-18–34) ‘We have the right as consumers to know what we eat, and that includes the virus information because we could eat it without knowing’ (P14-Belgium-Male-55+) ‘E-mail, it is easier because you can have a look any time’ (P43-Spain-Female-35–54) ‘Yes, I think that would be helpful. I think that they should have a media campaign on television and on the Internet” (P15-Belgium-Female-35–54) ‘From the government or the farmers since they are concerned by this. (P3-Belgium-Female-18–34) ‘The government [should provide information].. Because they will control and put rules on’ (P43-Spain-Female-35–54)	Government was the most frequently mentioned as preferred communication providers, followed by plant virus experts, farmers, minister of agriculture, and scientists
Which stakeholders are concerned about plant viruses	Farmers were thought to be the most worried stakeholders in relation to plant viruses because agriculture is the basis of the farmers’ livelihood	‘It’s their living anything that's going to affect somebody’s living is going to cause them to worry about it’ (P62-UK-Female-35–54). ‘Yes, because of the quantity of products, but not specifically for the virus. It is more about the money’ (P22-Slovenia-Male-18–34) ‘We eat the viruses, eat the products and have them too, which I think is not good for our health’ (P5-Belgium-Female-55+) ‘I am worried, because if there is a lot of viruses it means the increase in chemicals. I like to buy food without chemicals, locally produced’ (P22-Slovenia- Male-18–34) ‘…Some products could vanish, or the food price can increase to be much higher, like 2000%’ (P21-Slovenia-Male-35–54) ‘It’s not really the government who will suffer an economic impact. It is rather the farmers who go having to bear the consequences. […] So, I don't think government cares really, unless that really affects a lot of farmers at once which is a threat to the environment or a threat for them, for really a large number of farmers’ […] (P3-Belgium-Female-18–34) ‘No, people involved in the food chain are not worried because they don't care. They care only about the profit margins, so they will find another supplier’(P21-Slovenia-Male-35–54) ‘I was not worried until now, but after this conversation, yes. To be honest as I was not informed about this, I have never thought about viruses in plants’ (P58-Spain-Female-35–54) ‘I was not worried, but after this interview yes, a little because it’s a topic that I don’t know much about it’(P52-Spain-Male-18–34)	Farmers, as primary producers, where perceived to be most (negatively) affected by the occurrence of viruses in supply chains

3.

## Abbreviations

EFSA: European Food Safety Authority
EPPO: The European and Mediterranean Plant Protection Organization
EU: European Union
FAO: Food and Agriculture Organization
GMOs: Genetically modified organisms
NPPOs: National Plant Protection Organizations
PLH: Plant health
